# Immune infiltration phenotypes of prostate adenocarcinoma and their clinical implications

**DOI:** 10.1002/cam4.4063

**Published:** 2021-06-15

**Authors:** Zehua Ma, Xiankui Cheng, Ting Yue, Xun Shangguan, Zhixiang Xin, Weiwei Zhang, Jiahua Pan, Qi Wang, Wei Xue

**Affiliations:** ^1^ State Key Laboratory of Oncogenes and Related Genes Department of Urology Renji Hospital School of Medicine Shanghai Jiao Tong University Shanghai China; ^2^ Department of Pathology Shandong Provincial Hospital Affiliated to Shandong First Medical University Jinan Shandong Province China; ^3^ Department of Radiology Renji Hospital School of Medicine Shanghai Jiao Tong University Shanghai Shanghai China

**Keywords:** biological behaviors, genomic patterns, immune infiltration phenotypes, prognostic signature, prostate adenocarcinoma

## Abstract

**Background:**

Tumor‐infiltrating immune cells participate in the initiation and progression of prostate adenocarcinoma (PRAD). However, it is not fully known how immune infiltration affects the development of PRAD and its clinical presentation.

**Methods:**

Herein, we investigated the immune infiltration phenotypes in PRAD based on transcriptome profiles, methylation profiles, somatic mutation, and copy number variations. We also developed an immune prognostic model (IPM) to identify unfavorable prognosis. To verify this model, immunohistochemistry staining was performed on a cohort of PRAD samples. Moreover, we constructed a nomogram to assess the survival of PRAD incorporating immune infiltration and other clinical features.

**Results:**

We categorized PRAD patients into high and low‐level clusters based on immune infiltration phenotypes. The patients in the high‐level clusters had worse survival than their low‐level counterparts. Gene set enrichment analysis indicated that both anti‐ and pro‐tumor terms were enriched in high‐level cluster. Moreover, we identified a positive correlation between anti‐ and pro‐tumor immune cells in PRAD microenvironment. Notably, Somatic mutation analysis showed patients in high‐level cluster had a higher somatic mutation burden of KMT2D, HSPA8, CHD7, and MAP1A. In addition, we developed an IPM with robust predictive ability. The model can distinguish high‐risk PRAD patients with poor prognosis from low‐risk PRAD patients in both training and another three independent validation datasets. Besides, we constructed a nomogram incorporating Gleason score, pathological T stage, and IPM for the prognosis prediction of PRAD patients, which displayed robust predictive ability and might contribute to clinical practice.

**Conclusion:**

Our work illustrated the immune infiltration phenotypes strongly related to the poor prognosis of PRAD patients, and highlighted the potential of the IPM to identify unfavorable tumor features.

## Graphical abstract

Our work illustrated the immune infiltration phenotypes strongly related to the poor prognosis of PRAD patients and highlighted the potential of the immune prognostic model to identify unfavorable tumor features.

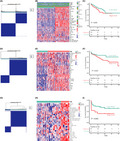

## 1 INTRODUCTION

Globally, prostate adenocarcinoma (PRAD) is the most common cancer in male populations and is ranked second in fatality.^1^ Currently, androgen‐deprivation therapy remains the primary treatment for patients with progressive and metastatic PRAD, yet most patients develop castration‐resistant prostate cancer after a period of androgen‐deprivation therapy, which is the leading cause of death for PRAD.^2^, ^3^ So far, clinicians determine treatment regimen and prognosis assessment for PRAD patients based on explicit clinicopathologic characteristics such as Gleason score, the TNM staging system, and prostate‐specific antibody (PSA) level, but current clinicopathologic markers have not been able to meet the growing demand of higher prediction accuracy of therapeutic efficacy and prognosis. Therefore, there is a need to explore other vigorous prognosis markers for PRAD patients who can benefit from current treatment.

As an important component of the tumor microenvironment, tumor‐infiltrating immune cells have been confirmed by numerous studies to participate in the development of PRAD.^4^, ^5^, ^6^ The relationship between tumor‐infiltrating immune cells and tumor is complex since besides self‐regulation, the composition and function of the former also influenced by PRAD’s development.^6^ Moreover, through various signaling pathways, cytokines, and remodeling microenvironment, tumor‐infiltrating immune cells can regulate the initiation and progression of PRAD by promoting crosstalk between tumor and stroma.^6^, ^7^, ^8^, ^9^ Researches also reported tumor‐infiltrating immune cells’ influence in the efficacy of clinical treatment.^10^, ^11^, ^12^ Immune checkpoint blockades (ICB) have significantly improved the prognosis of various tumor types,^13^ but they are not effective for castration‐resistant prostate cancer.^14^, ^15^ A recent study reported outstanding synergistic responses when combining ICB with MDSCs‐targeted therapy.^11^ However, the clinical significance of immune infiltration and the underlying mechanisms which mediate its participation in the development of PRAD remains poorly understood. Thus, we have the necessity to distinguish the immune signatures based on a comprehensive understanding of immune infiltration profiles and evaluated the predictive abilities of immune‐related responses for the prognosis of PRAD patients.

In the present study, we used “single sample Gene set enrichment analysis (ssGSEA)” to transform gene expression profiles into a relative abundance of 28 immune cells per individual tumor sample, based on specific gene sets of 28 immune cells. Before analyzing the association between clinicopathologic characteristics and immune infiltration phenotypes, PRAD patients were classified into two distinct phenotypes (low level and high level) based on the overall abundance of 28 tumor‐infiltrating immune cells in each PRAD sample. Our results showed that PRAD patients in high‐level cluster had worse clinicopathologic characteristics and prognosis compared with the low‐level group. We further studied the potential mechanisms underlying the role of immune infiltration phenotypes in the development of PRAD by analyzing the data of transcriptome profiles, methylation profiles, somatic mutation, and copy number variations (CNVs). Notably, our immune prognostic model (IPM), consisting of four immune cells (MDSC, pDC, T helper cells, Tgd, and Th1 cells), displayed robust predictive ability in both training and another three independent validation datasets. Additionally, besides demonstrating the suitability of this prognostic model in clinical decision making, our findings also suggest that related immune genes might act as potential therapeutic biomarkers for PRAD.

## 2 MATERIALS AND METHODS

### 2.1 Data acquisition from The Cancer Genome Atlas (TCGA)

The “Prostate Adenocarcinoma (TCGA, Firehorse Legacy)” dataset containing normalized mRNA expression data in RSEM (RNA‐seq by expectation maximization) and corresponding clinicopathologic information of 498 PRAD patients was obtained from cBioPortal (up to April 10, 2019).^16^ After log2(RSEM+1) transformation, mRNAs with less than 1 average expression values were excluded and low‐abundance profiles eliminated. Tumor purity, immune scores, and stromal scores were calculated using the ESTIMATE R package.^17^

The raw count of mRNA expression data of 498 PRAD patients was extracted from the FireBrowse database (http://www.firebrowse.org) (up to April 10, 2019). Genes with an average raw count of less than 1 were excluded, and low‐abundance profiles were eliminated.

The DNA methylation raw IDAT files of PRAD based on the Illumina Human Methylation 450k Array were downloaded using the TCGAbiolinks R package.^18^ We used the ChAMP R package to identify differentially methylated regions (DMRs) and their methylation levels were calculated using the average methylation levels of multiple CpG probes mapped to the DMRs.^19^

The somatic mutation profiles of PRAD based on the whole‐exome sequencing platform were downloaded using the TCGAbiolinks R package. Samples with missense mutations, frameshift insertions, frameshift deletions, in‐frame insertions, in‐frame deletions, nonsense mutations, multiple hits or splice‐site mutations were considered as mutation positives. For summarization and analysis of somatic mutation profiles, the maftools R package was used.^20^ Tumor mutation burden (TMB) was defined as the number of somatic, coding, base substitution, and indel mutations per megabase within the whole genome. In this study, 38 Mb was used as the exome size.^21^

Level 4 CNVs profiles of PRAD were downloaded from GDAC Firehose (http://gdac.broadinstitute.org) and divided into two distinct subtypes according to the immune infiltration phenotypes. GISTIC 2.0 was used to identify significant amplification or deletion alterations in the whole genome.^22^ In scoring, a locus with GISTIC value of more than 1 or less than −1 was defined as amplification or deletion, respectively.

### 2.2 Data acquisition from public database

The gene expression profile matrix files and corresponding clinicopathologic information from GSE70770 based on platform GPL10558 (including 203 PRAD samples) were downloaded from the GEO database (https://www.ncbi.nlm.nih.gov/geo/). The gene expression profile matrix files and corresponding clinicopathologic information from ICGC (including 105 PRAD samples) were downloaded from the cBioPortal (http://www.cbioportal.org). Where duplicate data were found, the average expression value was used. Genes with an average expression value less than 1 were excluded and low‐abundance profiles were eliminated.

### 2.3 Patients in the Renji PRAD cohort and sample collection

Participants in this study included 102 patients who were diagnosed with PRAD and underwent surgery between 2005 and 2014 at Renji Hospital, an affiliate of Shanghai Jiaotong University School of Medicine (Shanghai, China). All the selected participants did not undergo neoadjuvant therapy before surgery, and hematoxylin and eosin‐stained slides of each tumor sample were examined by two experienced pathologists. Final diagnosis was based on the morphology of the tumor samples after staining with H&E. Prior to sample collection, we obtained signed informed consent forms from all the patients who participated in the study. In addition, PRAD samples were collected from the 102 patients; fixed in formalin, and embedded in paraffin for the later examination of the infiltration levels of tumor‐infiltration immune cells.

### 2.4 Analysis of human TMAs

Specimens from radical prostatectomy were fixed in formalin and embedded in paraffin. A tissue core of approximately 1 mm in diameter containing a dominant tumor area was obtained from each specimen and arranged into a recipient block to form a tissue microarray. CD4 antibody (Maxim, MAB‐0740, 1:100), CD33 antibody (Abcam, ab11032, 1:100), Tbx21 antibody (Abcam, ab91109, 1:100), BDCA‐2 antibody (R&D Systems, AF1376, 1:200) or TCR γ/δ antibody (Biolegend, 118101, 1:20) was added to the slides followed by an overnight incubation at 4°C. The slides were then washed in PBS and incubated with appropriate peroxidase‐labeled goat anti‐rabbit IgG (H + L) secondary antibody (ab205718, 1:100, abcam) or goat anti‐mouse IgG (H + L) secondary antibody (ab205719, 1:100, abcam), for 60 min at 37°C. Each section was washed with PBS; developed with 3,3′‐diaminobenzidine solution; washed with water; and counterstained with hematoxylin. The degree of immunostaining and was scored independently by two clinically blind observers. The intensity of staining was scored as: 0 (negative), 1 (weak), 2 (moderate), and 3 (strong). The staining index (SI) was then calculated as staining intensity ×the proportion of positive cells.

### 2.5 ssGSEA

The relative abundance of tumor‐infiltrating immune cells was calculated using ssGSEA in the gsva R package. The ssGSEA transforms gene expression profiles into a relative abundance of immune cell populations in individual tumor samples based on specific gene sets of immune cells.^23^ These specific gene sets of immune cells were obtained from published articles.^24^, ^25^, ^26^, ^27^, ^28^, ^29^, ^30^ Based on the “ssGSEA matrix” and the ConsensusClusterPlus R package, consensus cluster analysis was conducted using the K‐means algorithm.^31^ The “ssGSEA matrix” was a matrix with ssGSEA scores of each immune cell in rows and sample ID in columns.

### 2.6 Differentially expressed genes (DEGs) analysis

Analysis of DEGs among two clusters in the TCGA cohort was conducted using the DESeq2 R package.^32^ Adjusted P‐value of each gene was calculated using the FDR method. An FDR of less than 0.05 and absolute log2‐fold change of more than 1 was set as the cut‐off for identifying DEGs.

### 2.7 GSEA

To evaluate the potential mechanism of immune infiltration phenotypes affecting tumor development, we performed GSEA analysis using clusterProfiler R package.^33^ The annotated gene set file (c2.all.v6.2.symbols.gmt and c5.all.v6.2.symbols.gmt) was selected as the reference gene set, and significance set at *p* < 0.05.

### 2.8 Functional enrichment analysis

Functional enrichment analysis was performed using the Metascape online tool (http://metascape.org).^34^

### 2.9 Construction and validation of an IPM

A total of 495 PRAD samples (279 low‐level cluster and 216 high‐level cluster patients) with both transcriptome profiles and survival information were selected for the following analyses. The detailed processes of how we constructed the prognostic model were as follows: 1. Based on the univariate Cox regression analysis, the prognostic significance of tumor‐infiltrating immune cells was evaluated, and those with *p *< 0.05 selected for further analyses; 2. Among tumor‐infiltrating immune cells with *p *< 0.05 in the univariate Cox regression analysis, LASSO‐Cox analysis was conducted to further reduce the candidates for the prognostic model. LASSO‐Cox analysis was repeated 1000 times using the glmnet R package and tumor‐infiltrating immune cells that were repeated more than 900 times selected as prognostic model related‐biomarkers; 3. A prognostic model was identified by extracting the regression coefficients from multivariate Cox regression analysis; 4. Finally, the risk scores of each patient were calculated by multiplying the relative infiltration level of each immune cell with its corresponding regression coefficient. The cut‐off value was calculated by the survminer R package (Version: 0.4.3, https://cran.r‐project.org/web/packages/survminer/index.html). The cut‐off value was then used to divide the PRAD patients into high and low‐risk subgroups. The log‐rank test and Kaplan–Meier survival analysis were used to evaluate the prognosis of these subgroups. To investigate the prognostic performance of the model, we conducted the time‐dependent ROC curve analysis using the time ROC R package, respectively. Besides, we validated the model's performance in predicting survival in another three independent validation datasets.

### 2.10 Construction and evaluation of the nomogram

The independent risk factors, validated by multivariate Cox regression analysis, were used to construct a nomogram for predicting survival probability for 1, 3, and 5 years. The rms R package was used to construct the nomogram and calibration plots which could evaluate the performance of the nomogram. The predictive accuracies of the nomogram and separate prognostic factors were also compared using the time‐dependent ROC curve analysis.

### 2.11 Statistical analysis

All statistical analysis was performed using R (v3.5.6). Between‐group comparisons of continuous variables were performed with the Mann–Whitney U‐test. Contingency table tests were performed with Fisher's exact test. Correlation was assessed with Spearman's correlation. All tests were two‐sided. The statistical significance level is set at 0.05.

## 3 RESULTS

### 3.1 Identification of the immune infiltration phenotypes in PRAD

**FIGURE 1:**
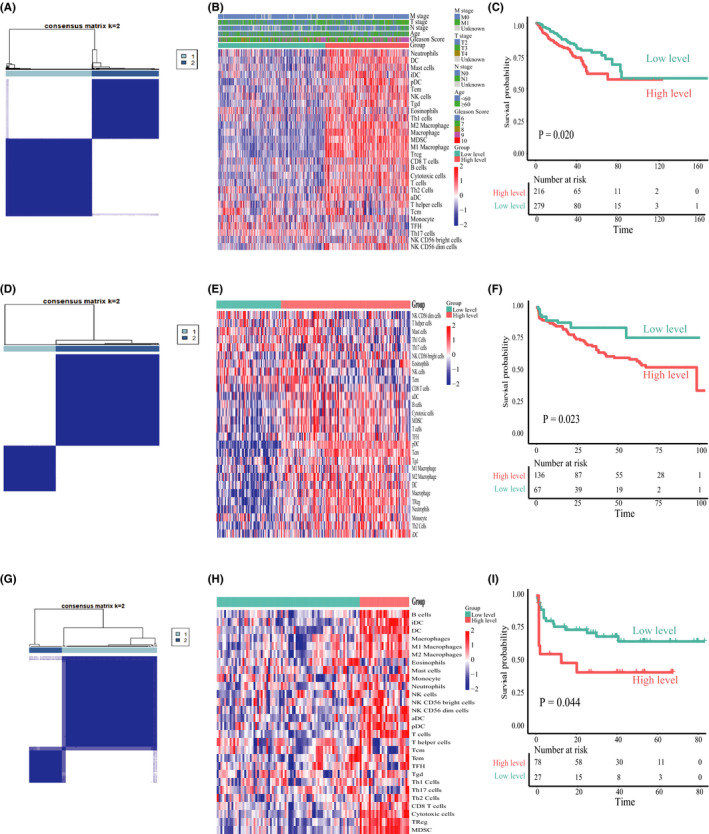
The immune infiltration phenotypes of prostate carcinoma. (A) Consensus clustering matrix of PRAD samples when K = 2 in the TCGA cohort. (B) Heatmap and clinicopathologic characteristics of two distinct immune infiltration phenotypes defined by the overall abundance of 28 tumor‐infiltrating immune cells in the TCGA cohort. (C) Kaplan–Meier survival curves of each cluster for DFS (disease‐free survival) in the TCGA cohort. (D) Consensus clustering matrix of PRAD samples when K = 2 in the GSE70770 cohort. (E) Heatmap of two distinct immune infiltration phenotypes defined by the overall abundance of 28 tumor‐infiltrating immune cells in the GSE70770 cohort. (F) Kaplan–Meier survival curves of each cluster for bPFS (Biochemical progression‐free survival) in the GSE70770 cohort. (G) Consensus clustering matrix of PRAD samples when K = 2 in the ICGC cohort. (H) Heatmap of two distinct immune infiltration phenotypes defined by the overall abundance of 28 tumor‐infiltrating immune cells in the ICGC cohort. (I) Kaplan–Meier survival curves of each cluster for bPFS (Biochemical progression‐free survival) in the ICGC cohort

To conduct a comprehensive evaluation of the immune infiltration in the PRAD microenvironment, we analyzed the three public genomic datasets from the public database. We started with analyzing public TCGA RNA‐Seq data. First, we used the gsva R package to transforms transcriptional profiles into the ssGSEA score of each immune cell in individual tumor samples based on specific gene sets of 28 immune cells. Then, the ConsensusClusterPlus R package was used to perform K‐means clustering on the “ssGSEA matrix.” The “ssGSEA matrix” was a matrix with ssGSEA scores of each immune cell in rows and sample ID in columns. Finally, according to the results of K‐means clustering analysis and the prognostic significance of different *K* values, *K* = 2 was selected as the optimal K value (Figure 1A and Figure S1). We then divided PRAD patients into two distinct phenotypes (low level and high level) and generated a heatmap to visualize the overall abundance of 28 tumor‐infiltrating immune cells of each phenotype (Figure 1B). To determine the correlation between immune infiltration phenotypes and prognosis, we performed the log‐rank test and Kaplan–Meier survival analysis, which revealed a significantly shorter disease‐free survival (DFS) in high‐level cluster than low‐level cluster (*p *= 0.020, Figure 1C). Further, we repeated the above analysis processes in another two independent cohorts from the public database and confirmed the classification of immune infiltration phenotypes, which also revealed a shorter biochemical progression‐free survival (bPFS) in high‐level cluster (Figure 1D‐I and Figure S1). In addition, we investigated the association between clinicopathologic features and immune infiltration phenotypes (Table 1). In the recent PSA level, pathological N stage, and clinical M stage, there was no significant statistical difference observed between the two clusters (all *p *> 0.05). However, there were significantly higher Gleason score and pathological T stage in the high‐level cluster than low‐level cluster (*p *= 0.0066 and *p *= 0.0093, respectively). To explore the underlying relationships between different tumor‐infiltrating immune cells, we calculated the Pearson's correlation coefficients among tumor‐infiltrating immune cells. Figure S2A showed the strongest positive correlation existed between Treg, M1 Macrophage, and MDSCs. The tumor microenvironment includes not only the immune cells, but also the tumor cells, fibroblasts, and other cells, as well as the intercellular substance, capillaries, and biomolecules infiltrated in it. Since plentiful studies have revealed that tumor purity can affect the prognosis of tumor patients,^35^, ^36^ we used the ESTIMATE R package to compare the tumor composition between two clusters. Tumor purity was higher in low‐level cluster (*p *< 0.0001, Figure S2C), but we found tumor purity was not associated with the prognosis of PRAD patients (*p *= 0.26, Figure S2D). Meanwhile, we also found the immune and stroma scores were higher in high‐level cluster (*p *< 0.0001, Figure S2B), which indicated tumor‐infiltrating immune cells may interact with other components of the tumor microenvironment. Above all, these findings suggest that PRAD samples with high immune infiltration level have worse prognosis than their low counterparts. This cluster is characterized by intensive local immune responses and lower tumor purity, and enriched with some pro‐tumor immune cells such as Treg and MDSCs.

**TABLE 1:** Demographic and clinicopathologic characteristics of PRAD patients from the TCGA cohort between the two clusters

	Low‐level cluster	High‐level cluster	*p*‐value
Number of patients	280	218	
Recent PSA (mean (sd))	2.12(20.59)	1.26(4.56)	0.5648
Age (%)			0.0622
<60	125(44.64)	77(35.32)	
≥60	155(55.36)	141(64.68)	
Gleason score (%)			0.0066
6–7	179(63.93)	113(51.83)	
8–10	101(36.07)	105(48.07)	
Pathologic_T (%)			0.0093
T2	119(43.12)	68(31.63)	
T3	154(55.80)	139(64.65)	
T4	3(1.08)	8(3.72)	
Pathologic_N (%)			0.1170
N0	193(83.91)	152(77.95)	
N1	37(16.09)	43(22.05)	
Clinical_M (%)			0.7086
M0	255(99.22)	201(99.50)	
M1	2(0.78)	1(0.50)	

### 3.2 Potential role of immune infiltration phenotypes in PRAD

**FIGURE 2:**
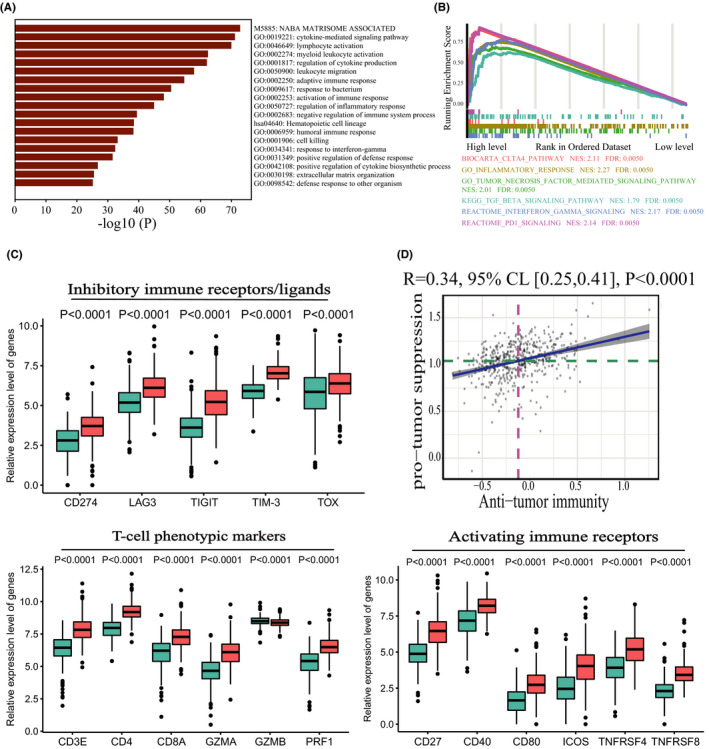
Enrichment analysis of the immune infiltration phenotypes. (A) Functional enrichment analysis indicates the significant biological processes enriched in the high‐level cluster. (B) Gene set enrichment analysis indicates significant signaling pathways correlated with the immune infiltration phenotypes using gene sets of “c2.all.v6.2.symbols” and “c5.all.v6.2.symbols.” (C) Differential expression of immune‐related genes between the two clusters. (D) Positive Spearman's correlation of the infiltration level between immune cells executing anti‐tumor immunity (Tcm, Tem, Th1 cells, Th17 cells, cytotoxic cells, aDC, and NK CD56 bright cells) and immune cells executing pro‐tumor suppression (Treg, Th2 cells, NK CD56 dim cells, pDC, Macrophage, MDSCs, and neutrophils). The shaded area represents a 95% confident interval

To further discuss the relationship between transcriptional profiles and immune infiltration phenotypes, we reanalyzed the TCGA RNA‐Seq data. Principal component analysis revealed that the transcriptional profiles differed between two clusters with the results indicating distinct biological behaviors between them (Figure S3). Our analysis revealed 1287 upregulated and 57 downregulated DEGs in the high‐level cluster in contrast to low‐level cluster (Figure S4). To annotate the biological functions of these DEGs, we performed functional enrichment analysis using the Metascape online tool. As illustrated in Figure 2A, the results revealed that upregulated DEGs in high‐level cluster were significantly enriched in immune‐related biological processes and signaling pathways. To evaluate the potential mechanism of immune infiltration in tumor development, we performed GSEA analysis and the results showed that both pro‐tumor and anti‐tumor pathways were enriched in high‐level cluster such as: BIOCARTA_CTLA4_PATHWAY; REACTOME_PD1_SIGNALING; KEGG_TGF_BETA_SIGNALING_PATHWAY; GO_TUMOR_NECROSIS_FACTOR_MEDIATED_SIGNALING_PATHWAY; GO_INFLAMMATORY_RESPONSE; and REACTOME_INTERFERON_GAMMA_SIGNALING (Figure 2B and Table S1). Similar to the results of GSEA analysis, GZMA, PRF1 and IFNG showed significantly higher expression in high‐level cluster (*p *< 0.0001, Figure 2C). GZMA, PRF1, and IFNG are key cytolytic effectors which are significantly overexpressed upon the activation of CD8+ T cell or during productive clinical responses to ICB.^23^, ^37^, ^38^, ^39^ T‐cell phenotypic markers (e.g., CD3E, CD4, and CD8A) and activating immune receptors (e.g., CD27, CD40, CD80, and ICOS) also had significantly higher expression level in high‐level cluster (*p *< 0.0001, Figure 2C). Moreover, inhibitory immune factors (e.g., CD274, LAG3, TIGIT, TIM‐3, and TOX) showed higher expression in high‐level cluster (*p *< 0.0001, Figure 2C). According to the generated heatmap in Figure 1B, we observed that the two clusters had totally heterogeneous compositions of immune cells. Interestingly, high‐level cluster recruited not only immune cells executing anti‐tumor reactivity but also immune cells delivering pro‐tumor reactivity. Pearson's correlation analysis further revealed a significant positive correlation between these two categories of immune cells within the tumor microenvironment in PRAD (Figure 2D). By demonstrating the tumor microenvironment in PRAD, these findings suggest the presence of a potential feedback mechanism that may induce the infiltration of immunosuppressive cells and activation of pro‐tumor immune responses by sensing the activation of anti‐tumor immune responses.

### 3.3 Genomic characteristics of differential methylation profiles between two clusters

**FIGURE 3:**
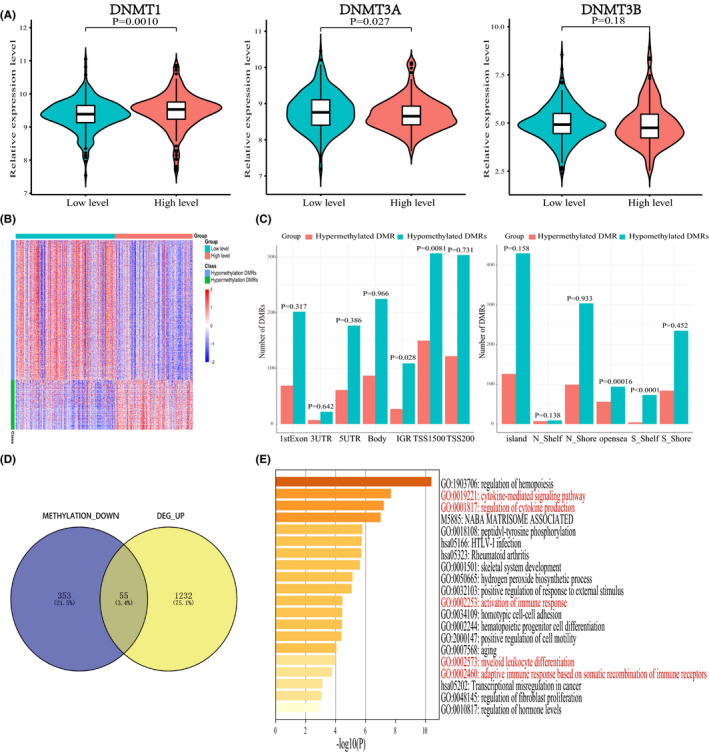
Association between immune infiltration phenotypes and DNA methylation patterns in PRAD. (A) Differential expression of three DNA methyltransferases between the two clusters. (B) Heatmap of 784 DMRs (Differentially methylated regions) between the two clusters. (C) Distribution of DMRs around the islands and on gene's different structural regions. (D) The intersection results of upregulated DEGs (Differentially expressed genes) and genes with decreased CpG island methylation level in the promoter region. (E) Enrichment analysis of the intersection results in (D)

DNA methylation is an important epigenetic modification mechanism that plays a crucial role in the regulation of gene expression and other physiologic and pathological processes.^40^ Therefore, we analyzed the PRAD samples with available methylation profiles in the TCGA cohort. DNA methyltransferases are mainly composed of DNMT1, DNMT3A, and DNMT3B.^41^ Compared with low‐level cluster, DNM1 displayed higher expression in high‐level cluster (*p *= 0.0010, Figure 3A), while DNM3A showed lower expression in high‐level cluster (*p *= 0.027, Figure 3A). Out of the 783 DMRs identified using the ChAMP R package, 578 DMRs were hypomethylated regions, while 205 DMRs were hypermethylated regions in the high‐level cluster in contrast to low‐level cluster. The normalized methylation levels of the 783 DMRs in the two clusters were presented in Figure 3B. We then investigated the position distributions around CpG islands and on different structural fragments for genes bearing the 783 DMRs with the results indicating that more hypermethylated DMRs located in TSS1500 (*p *= 0.0081, Figure 3C) and opensea (*p *= 0.00016, Figure 3C), but more hypomethylated DMRs were located in the intergenic region (*p *= 0.028, Figure 3C) and S_Shelf (*p *< 0.0001, Figure 3C). Since the methylation of CpG islands in the promoter region is usually related to the silencing of the corresponding gene,^42^ we compared the upregulated DEGs to genes with decreased methylation level of CpG islands in the promoter region, and as shown in Figure 3D, 55 genes were revealed. By annotating their biological functions, a significantly enhanced immune response‐phenotype was revealed in high‐level cluster (Figure 3E).

### 3.4 Analysis of somatic mutations and CNVs in patients with distinct immune infiltration phenotype

Since somatic mutations and CNVs can significantly affect the tumor characteristics and biological behaviors, we analyzed the PRAD samples with available somatic mutation and CNVs information to discover the potential molecular mechanisms influencing immune infiltration phenotypes within PRAD in the TCGA cohort. TMB, an emerging biomarker of ICB responses, displayed no significant difference between the two clusters (*p *= 0.34, Figure S5A). To further explore the relationship between TMB and the efficacy of ICB, we selected a gene panel as the biomarker to reflect the infiltration level of effector T‐cell (CD8A, CXCL9, and CXCL10) and IFN‐γ‐associated cytotoxicity (IFNG, GZMA, GZMB, EOMES, and TBX21).^43^ Besides, the expression level of this gene panel showed a strong positive correlation with anti‐PDL1 immunotherapy efficacy.^43^ There was, however, no significant association found between the expression level of the POPLAR biomarker panel and the TMB (Figure S5B), with the results indicating a weak association between TMB and cytolytic activity.

**FIGURE 4:**
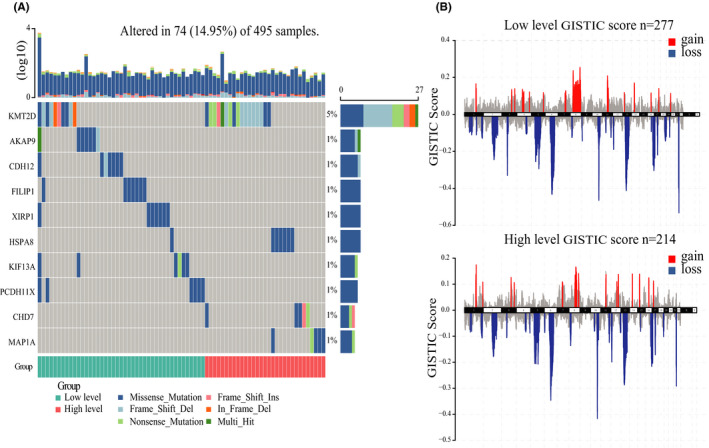
Association between immune infiltration phenotypes and somatic mutations and CNVs in PRAD. (A) Significantly differentially mutated genes between the two clusters. (B) Composite copy number profiles of high‐level cluster compared with low‐level cluster with gains shown in red and losses in blue

In the aspect of somatic mutation frequency, patients in the high‐level cluster had a higher frequency of somatic mutations in KMT2D (Figure 4A and Table S2), which has been reported to sustain prostate carcinogenesis and metastasis.^44^, ^45^ Although current studies have barely investigated their roles in PRAD, higher frequencies of somatic mutations were also observed in HSPA8, CHD7, and MAP1A (Figure 4A and Table S2).

Subsequently, we evaluated different chromosomal alteration patterns between the two clusters using GISTIC2.0. We found a total of 602 genes with different amplification frequencies and another 802 genes with different deletion frequencies in the high‐level cluster, compared with the low‐level cluster (Table S3). 10q26.13 encompassing FGFR2 locus was the most significantly deleted genomic region (71.43% in high‐level cluster, *p *= 0.0029, Figure 4C and Table S2). 12q21.31 encompassing ACSS3/has‐mir‐617/has‐mir‐618/LIN7A/MIR4699/MIR617/MIR618/PPFIA2 locus was the most significantly amplified genomic region (85.71% in high‐level cluster, *p *= 0.0017, Figure 4B and Table S3).

### 3.5 Construction of an immune prognostic model (IPM)

**FIGURE 5:**
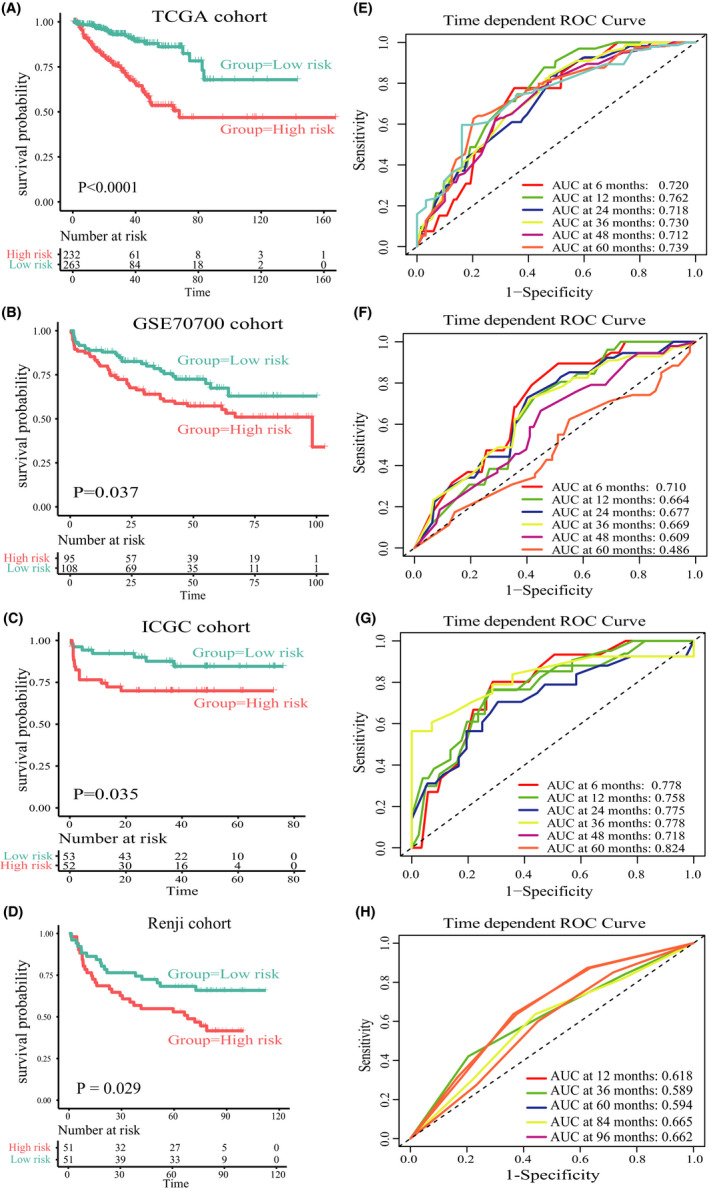
Prognostic analysis of the IPM (Immune prognostic model) in three independent cohorts. (A–D) Kaplan–Meier survival analysis was performed to compare prognosis between high‐risk score and low‐risk score subgroup in the TCGA, GSE70770, ICGC, and Renji cohorts. (E–H) Time‐dependent ROC curve analysis was performed to evaluate the predictive performance of the IPM in the TCGA, GSE70770, ICGC, and Renji cohorts

According to the above results, we could conclude that high‐level cluster was characterized by various tumor‐infiltrating immune cells. Therefore, the complex and comprehensive impacts generated by the interaction of various immune cells may be related to the poor prognosis of high‐level cluster. First, in order to determine the influence of tumor‐infiltrating immune cells on the prognosis of PRAD, we investigated the predictive ability of tumor‐infiltrating immune cells on the prognosis of PRAD. Univariate Cox regression analysis revealed that 6 of the 28 tumor‐infiltrating immune cells were significantly related to the prognosis of PRAD (Table S4). Then, we applied L1‐penalized Lasso regression analysis and subsequent multivariate Cox regression analysis to select immune cells with the greatest prognostic value, and finally selected MDSCs, Th1 cells, T helper cells, Tgd, and pDC. The regression coefficient of each tumor‐infiltrating immune cell was obtained from the multivariate Cox analysis. Finally, by weighting the relative infiltrating level of each tumor‐infiltrating immune cells to its regression coefficient, we established an IPM to predict the prognosis of PRAD patients (risk score = 0.6769 × relative infiltrating level of MDSC + 0.6472 × relative infiltrating level of pDC + 0.5369 × relative infiltrating level of T helper cells−0.6389 × relative infiltrating level of the Tgd–0.8783 × relative infiltrating level of Th1 cells). We calculated the cut‐off value (0.34) using the survminer R package. Then, the cut‐off value was used to classify the patients into high and low‐risk subgroups. As illustrated in Figure 5A, patients in the high‐risk subgroup had a much shorter DFS than patients in the low‐risk subgroup (HR: 3.74; 95% CI: 2.35–5.95; *p *< 0.0001). In addition, we verified the performance of the IPM in predicting prognosis, and the area under the ROC curve (AUC) was 0.720 at 6 months, 0.762 at 12 months, 0.718 at 24 months, 0.730 at 36 months, 0.712 at 48 months, 0.739 at 60 months (Figure 5E). The overall AUC and C‐index of the IPM were 0.71 and 0.71, respectively (Table S5). Besides, both univariate and multivariate Cox analyses implied that the performance of IPM in predicting DFS is independent of immune infiltration phenotypes (Figure S6).

### 3.6 Evaluation and validation of the IPM in three validation cohorts

To further evaluate the robustness and practicality of the IPM, we examined the model's performance in predicting biochemical progression‐free survival in two independent validation cohorts. The two cohorts had 203 and 105 PRAD patients, respectively. The cut‐off value (0.34) was used to classify patients of each cohort into high and low‐risk subgroups. Similar to the TCGA cohort results, the patients in the high‐risk subgroup had a much worse prognosis than patients who were assigned to the low‐risk subgroup in three independent validation cohorts (GSE70770: HR: 1.66, 95% CI: 1.03–2.68, *p *= 0.037, Figure 5B; ICGC: HR: 2.55, 95% CI: 1.04–6.26, *p *= 0.035, Figure 5C; Renji: HR: 1.93, 95% CI: 1.06–3.51, *p *= 0.029, Figure 5D). In GSE70770, the AUC was 0.710 at 6 months, 0.664 at 12 months, 0.677 at 24 months, 0.669 at 36 months, 0.609 at 48 months, 0.486 at 60 months (Figure 5F), the overall AUC and C‐index were 0.63 and 0.62, respectively (Table S5). In ICGC, the AUC was 0.778 at 6 months, 0.758 at 12 months, 0.775 at 24 months, 0.778 at 36 months, 0.718 at 48 months, 0.824 at 60 months (Figure 5G), the overall AUC and C‐index were 0.74 and 0.73, respectively (Table S5). Further, in Renji cohort, the area under the ROC curve was 0.618 at 12 months, 0.589 at 36 months, 0.594 at 60 months, 0.665 at 84 months, 0.662 at 96 months (Figure 5H), the overall AUC and C‐index were 0.65 and 0.65, respectively (Table S5). These results indicated that the IPM had a robust performance in distinct cohorts.

### 3.7 Altered biological behaviors in high‐risk and low‐risk subgroups

**FIGURE 6:**
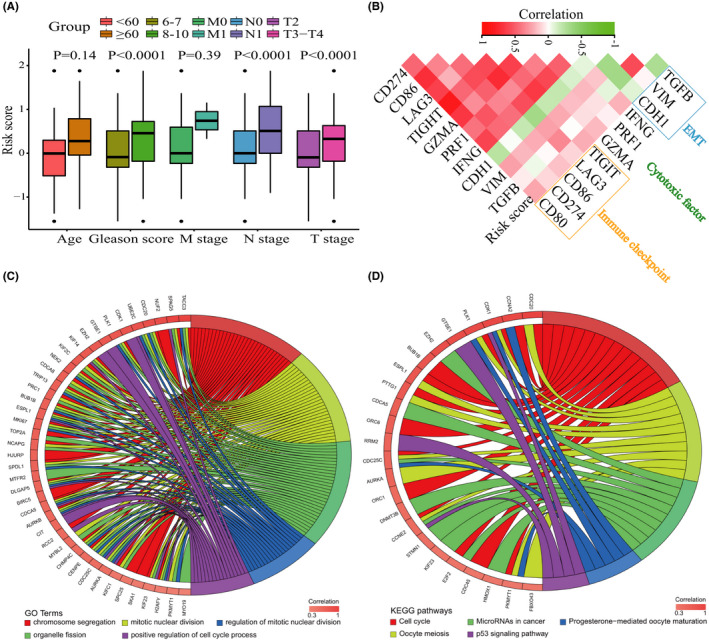
Clinicopathologic significance and biological function of the immune infiltration‐based prognostic model. (A) Risk scores in different clinicopathologic subgroups. (B) Correlation matrix of risk scores and the expression levels of certain genes. The color of the square reflects the corresponding correlation coefficients. (C) Circular plot of the enriched biological processes of the risk score associated genes. (D) Circular plot of the enriched KEGG pathways of the risk score associated genes

We further evaluated the associations between risk scores and clinicopathologic characteristics, and the results showed that risk scores significantly varied between PRAD patients with different Gleason scores, pathologic N stages, and pathologic T stages (*p <* 0.0001, Figure 6A). As illustrated in Figure 6B, the risk scores showed significant positive correlation with the expression of CD274 (*p <* 0.0001), CD80/86 (*p <* 0.0001), LAG3 (*p <* 0.0001), TIGIT (*p <* 0.0001), GZMA (*p <* 0.0001), PRF1 (*p <* 0.0001), and IFNG (*p <* 0.0001). Conversely, they revealed negative correlation with markers of epithelial–mesenchymal transition (EMT). Further, we performed GO and KEGG analysis to evaluate the potential biological behaviors associated with the immune infiltration‐based prognostic model. Genes whose expression had a strong positive correlation with risk score (Pearson correlation coefficient >0.3 and *p <* 0.05) were considered as risk score‐associated genes. Based on GO analysis, these genes were significantly enriched in: chromosome segregation; mitotic nuclear division; regulation of mitotic nuclear division; organelle fission; and positive regulation of cell cycle process (Figure 6C). In addition, based on KEGG analysis, they were enriched in: Cell cycle; MicroRNAs in cancer; Progesterone‐mediated oocyte maturation; Oocyte meiosis; and p53 signaling pathway (Figure 6D).

### 3.8 The IPM is independent of conventional clinicopathologic characteristics

**FIGURE 7:**
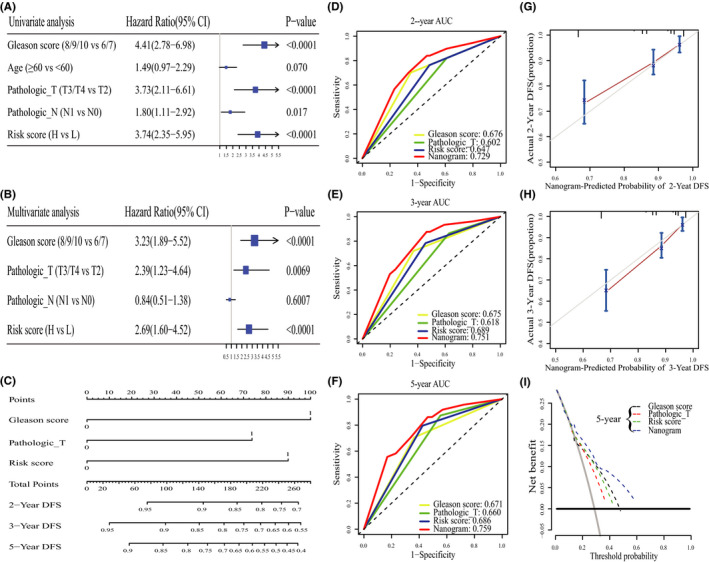
Relationship between the IPM and other clinical information. (A) Univariate regression analysis of the relationship between the IPM and clinicopathological characteristics associated with DFS in the TCGA cohort. (B) Multivariate regression analysis of the relationship between the IPM and clinicopathological characteristics associated with DFS in the TCGA cohort. (C) Nomogram constructed to predict the 2‐, 3‐, and 5‐year DFS for PRAD patients. (D–F) Time‐dependent ROC curve analyses of Gleason score, pathologic T stage, risk score, and the nanogram. (G–H) Calibration curve of the nomogram for predicting the probability of DFS at 2 and 3 years. (I) Decision curve analyses of Gleason score, pathologic T stage, and the nomogram for 5‐year risk

We performed univariate and multivariate Cox analyses to determine the prognosis independence of the IPM from other conventional clinicopathologic characteristics. After adjusting for conventional clinicopathologic characteristics, including age, pathologic T stage, pathologic N stage, and Gleason score, the IPM still operated as an independent prognostic factor. This, therefore, confirmed its robust ability in predicting prognosis in PRAD (Figure 7A, B). Further, the IPM had the second highest mean C‐index (0.65, Table S6) when compared with conventional clinicopathologic characteristics. These results indicated that the IPM has a robust predictive ability for prognosis, and is independent of conventional clinicopathologic characteristics.

### 3.9 Construction and evaluation of a nomogram

To provide a quantitative approach to predicting the prognosis of PRAD patients for clinicians, we developed a nomogram by integrating the IPM with classical clinical risk factors (pathological T stage and Gleason score) (Figure 7C). Based on the multivariate Cox analysis, points were assigned to each variable using the point scale of the nomogram, and the points for each patient were determined by summing the points of all variables. The estimated DFS rate of PRAD patients at 2 years, 3 years, and 5 years were calculated by drawing a vertical line from the total point coordinate axis down to the prognostic coordinate axis. The nomogram revealed that the IPM had the second highest weight among the variables, which was consistent with the multivariate Cox analysis results. Moreover, our nomogram recorded a C‐index of 0.72 with 1000 bootstrap iterations (95% CI: 0.69–0.75, Table S6). We further compared the predictive ability of the nomogram with that of the Gleason score, pathologic T stage, and the IPM. The results showed that nomogram (C‐index: 0.72) was better than Gleason score (C‐index: 0.68), pathologic T stage (C‐index: 0.55) and the IPM (C‐index: 0.65). Besides, the nomogram also had the largest 2‐year AUC, 3‐year AUC, and 5‐year AUC (Figure 7D–F). The calibration plots indicated that the bias‐corrected line was close to the ideal curve, indicating a good consistency between the nomogram‐predicted outcomes and the actual outcomes (Figure 7G, H). Similarly, based on the decision curve, the nomogram had a higher net benefit than the Gleason score, pathological T stage, and immune infiltration‐based prognostic model (Figure 7I). Consequently, these results indicated that our nomogram had a better predictive ability for predicting the prognosis of PRAD patients than other conventional clinicopathologic factors.

## 4 DISCUSSION

Although extensive researches indicate that the immune microenvironment plays a vital role in the initiation and progression of tumors, the current understanding of the relationship between tumors and immune microenvironment is not sufficient to influence the clinical treatment of tumors. Besides, as an effective and promising treatment option, the clinical benefit of ICB for PRAD patients is particularly limited. Şenbabaoğlu et al. applied unsupervised clustering to classify the immune microenvironment of clear cell renal cell carcinoma patients into three clusters by immune infiltration scores, and found immune infiltration correlated with the prognosis and efficacy of ICB.^46^ However, few studies have explored immune infiltration phenotypes of PRAD. In this study, unsupervised consensus clustering of PRAD patients using overall immune infiltration levels revealed two distinct phenotypes of differentially infiltrated PRAD patients; high‐level and low‐level clusters. The high‐level cluster correlated with worse prognosis as validated by another two independent GEO cohorts. The patients in high‐level cluster also presented more malignant clinicopathological characteristics and worse prognosis. In the aspect of tumor purity, high‐level cluster had lower tumor purity than low‐level cluster, but we did not find that tumor purity was associated with the prognosis of PRAD patients. The results indicated tumor purity was not the cause of the poor prognosis of the high‐level cluster. These results provided a “bridge” connecting the immune infiltration and PRAD characteristics, and illustrated that the dynamic changes of immune infiltration in the microenvironment could affect PRAD’s features and biological behaviors.

During tumor development, several immunosuppressive mechanisms can drive the progression and metastasis of tumors such as high infiltration level of immunosuppressive cells, high expression level of immunosuppressive factors, and evasive anti‐tumor immune responses.^47^ For this reason, we investigated the immune microenvironment of PRAD patients in high‐level cluster. We found that both anti‐ and pro‐tumor pathways were enriched in high‐level cluster as revealed by the GSEA analysis results. In addition, both key cytolytic effectors (GZMA, PRF1, and IFNG) and immunosuppressive factors (CD274, LAG3, TIGIT, TIM‐3, and TOX) showed higher expression in high‐level cluster. Besides, we also identified a positive correlation between anti‐ and pro‐tumor immune cells in PRAD patients. These results indicated a positive feedback regulatory mechanism in the immune microenvironment that drives the development of PRAD tumors.

Evidence from previous studies show that TMB is an independent predictor of the efficacy of ICB.^48^, ^49^ We found that the expression of TMB was relatively low in PRAD patients, and this may partially explain the suboptimal efficacy of ICB in PRAD patients. Higher TMB is associated with stronger anti‐tumor immune responses, but the present study found no significant correlation between TMB level and the strength of anti‐tumor immune responses in PRAD patients. Different mutational processes generally produce distinct combinations of mutation types. Therefore, we compared the mutation signatures between the two clusters, but found no difference. Mutation analysis showed that PRAD patients in high‐level cluster had a higher frequency of somatic mutations in KMT2D, HSPA8, CHD7, and MAP1A. Among these genes, KMT2D, a major mammalian histone H3 lysine 4 mono‐methyltransferase, sustains prostate carcinogenesis and metastasis. Our findings show that somatic mutations in KMT2D, HSPA8, CHD7, and MAP1A are related to the immune infiltration phenotype of PRAD. These four genes may be involved in regulating the immune infiltration of PRAD, but this requires further experimental verification. Subsequent research may focus on the functions of these four genes and explore whether and how these four genes affect the immune infiltration of PRAD.

Given that tumor‐infiltrating immune cells are significantly correlated with the prognosis of PRAD patients, we constructed an IPM for personalized prognostic prediction based on five tumor‐infiltrating immune cells, MDSCs, Th1 cells, T helper cells, Tgd, and pDC. This model was able to identify PRAD patients with poor prognosis. The robustness of this model was then validated by another three independent cohorts. The five selected tumor‐infiltrating immune cells could individually serve as therapeutic targets for PRAD cancer. Moreover, ICB combined with targeted therapy against MDSCs has been reported to exhibit robust synergistic efficacy in both primary and metastatic CRPC. Currently, no study has reported whether each of the other four immune cells can be individually used as a biomarker to monitor the clinical response to cancer treatments. Although regulatory T cell was not included in the prognostic model, it has been reported that prostate tumor‐bearing mice showed delayed development of castration resistance and better prognosis when combining ADT with Treg depletion in contrast to ADT alone.

Further analysis revealed that the IPM remained as an independent prognostic factor after adjustment for clinicopathologic characteristics. Moreover, we built a comprehensive nomogram by merging our model with other clinicopathologic characteristics (pathological T stage and Gleason score). Assessment of the nomogram using a ROC curve and calibration plot, showed that it could accurately predict the 2‐, 3‐, and 5‐year DFS. A key advantage of this model is that it provides a quantifiable individual scoring system for identifying patients with high risk of poor prognosis. Consequently, our nomogram may be applied in clinical practice in the future with further modifications. Moreover, since the rapid development of high‐throughput sequencing technology, we strongly believe our IPM and nomogram has the potential to be applied into clinical application. Besides the composition of tumor‐infiltrating immune cells, the distribution of tumor‐infiltrating immune cells has also played an important role in the anti‐tumor immune response. However, few studies focus on the relationship between the distribution of tumor‐infiltrating immune cells and the prognosis of PRAD. Future research on the relationship between the distribution of tumor‐infiltrating immune cells and the prognosis of PRAD may further improve the performance of the immune prognostic prediction model.

This study provides some new insights into the impact of immune infiltration on the development of PRAD. However, some limitations in our study are worth highlighting. First, clinical information on the public databases is limited, thus the clinicopathologic characteristics used in this study are not comprehensive, and this might decrease the reliability and practicality of the established nomogram. Second, this is a retrospective study, thus our results require further validation through prospective studies. Third, although we calculated the cutoffs in this article, more appropriate cutoffs should be defined in future clinical studies.

## 5 CONCLUSION

To the best of our knowledge, this is the first study to classify PRAD patients into two distinct clusters based on their respective immune infiltration phenotypes and to establish an immune prognostic model regarding survival for PRAD patients. In addition, to further understand the relationship between PRAD and immune infiltration, we explore the potential role of immune infiltration phenotypes in PRAD from different perspectives including transcriptome profiles, DNA methylation profiles, somatic mutation, and copy number variations. The prognostic model presented herein has good and independent predictive ability, with a nomogram incorporating Gleason score, pathologic T stage, and the immune prognostic model for clinical application in the prognosis prediction of PRAD patients.

## 6 ETHICS STATEMENT

The use of pathological specimens and the review of all pertinent patient records were approved by the Shanghai Jiao Tong University School of Medicine, Renji Hospital Ethics Committee (RA‐2019–241). All participants provided written informed consent for the use of their samples for research purposes.

## CONFLICT OF INTEREST

The authors declare that there are no conflicts of interest.

## AUTHOR CONTRIBUTIONS

All authors searched the literature, designed the study, collected the specimen, collected the clinical information, and revised the manuscript. ZM, XC, and TY completed most of the analyses and experiments; SG, ZX, and WZ conducted a specific subset of the analyses and experiments; ZM and QW wrote the manuscript. All authors reviewed and approved the final manuscript.

## Supporting information

Fig S1

Fig S2

Fig S3

Fig S4

Fig S5

Fig S6

Table S1

Table S2

Table S3

Table S4

Table S5

Table S6

## Data Availability

All the public datasets can be downloaded from the cBioPortal and GEO database. All other data supporting the findings of this study are available from the corresponding author upon reasonable request.
