# Supplementary material for: Immune infiltration phenotypes of prostate adenocarcinoma and their clinical implications
Source: Cancer Med. 2021 Jun 15;10(15):5358–74. doi: 10.1002/cam4.4063 (PMC8335836; doi:10.1002/cam4.4063)
Supplement: Supplementary file 2 — Fig S2 [file CAM4-10-5358-s012.pdf]

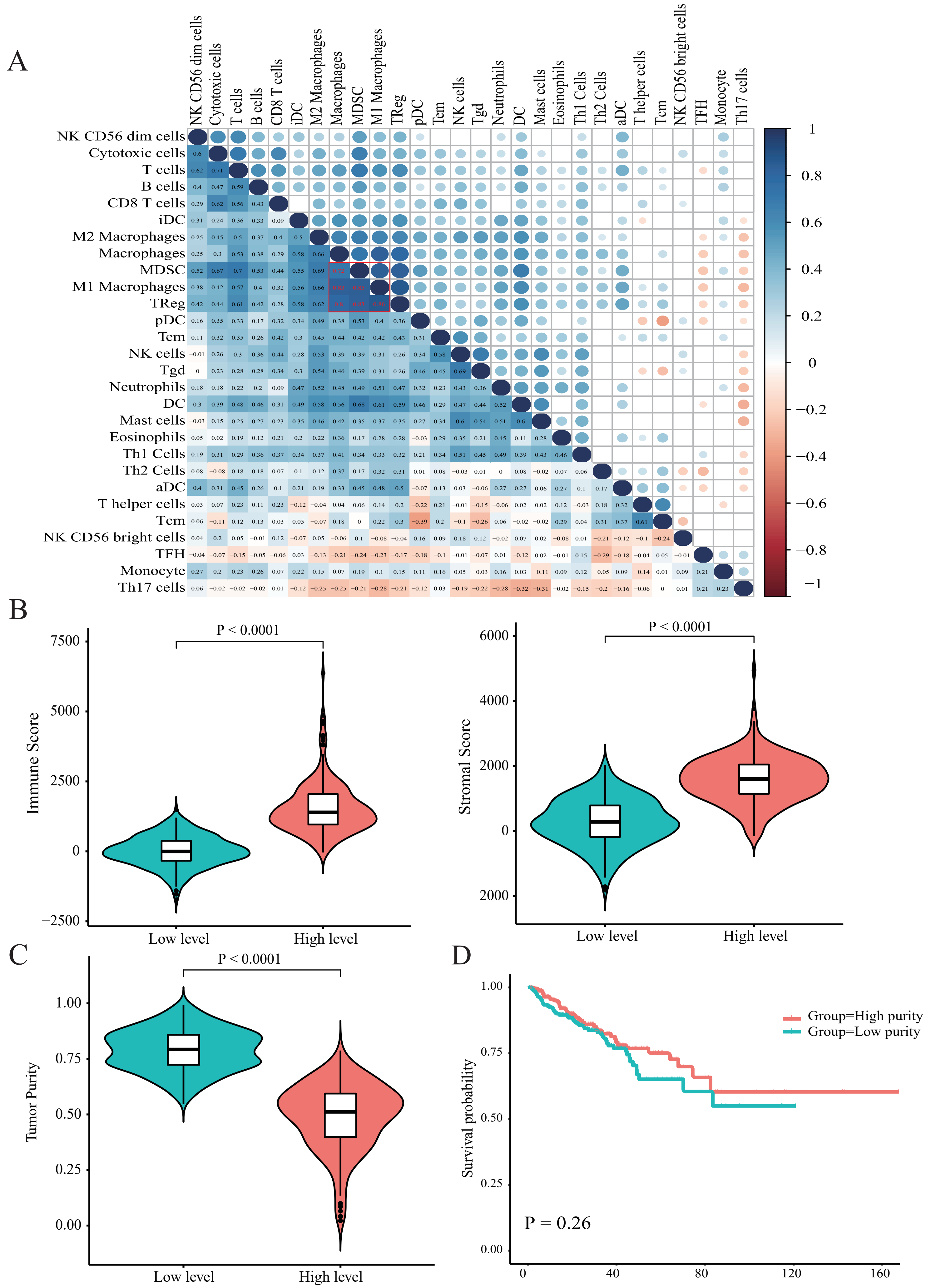

Supplementary Fig. 2 (A) Correlation matrix of all the 28 detected tumor-infiltrating immune cell types in the TCGA cohort. (B) Distinct distribution of immune score and stromal score between the two clusters. (C) Distinct distribution of tumor purity between the two clusters. (D) Kaplan-Meier survival curves of tumor purity for DFS in the TCGA cohort.
