# Supplementary material for: Immune infiltration phenotypes of prostate adenocarcinoma and their clinical implications
Source: Cancer Med. 2021 Jun 15;10(15):5358–74. doi: 10.1002/cam4.4063 (PMC8335836; doi:10.1002/cam4.4063)
Supplement: Supplementary file 3 — Fig S3 [file CAM4-10-5358-s004.pdf]

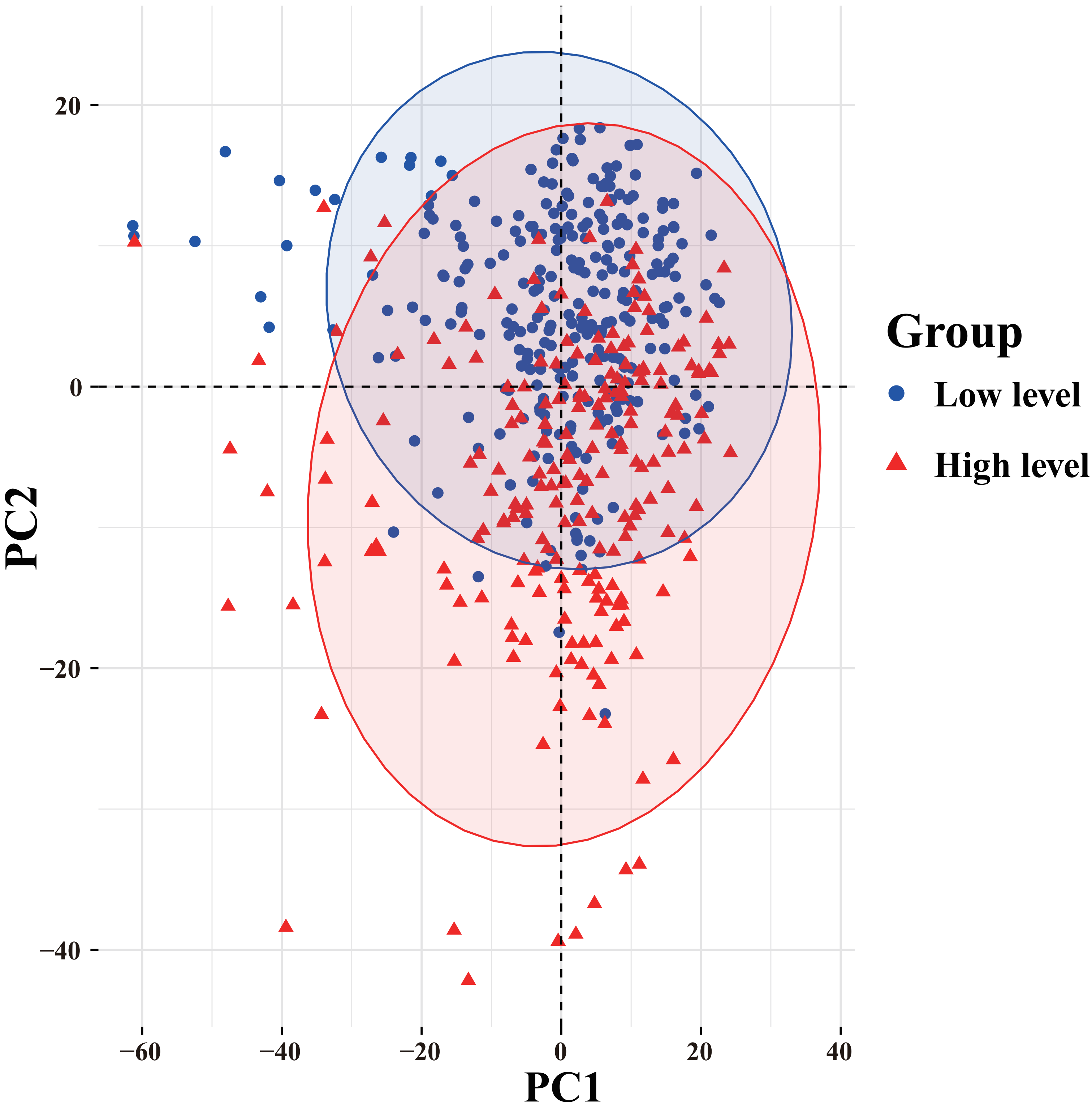

Supplementary Fig. 3 Principal component analysis was performed based on transcriptomic expression profiles of PRAD patients from two clusters.
