# Supplementary material for: Immune infiltration phenotypes of prostate adenocarcinoma and their clinical implications
Source: Cancer Med. 2021 Jun 15;10(15):5358–74. doi: 10.1002/cam4.4063 (PMC8335836; doi:10.1002/cam4.4063)
Supplement: Supplementary file 5 — Fig S5 [file CAM4-10-5358-s006.pdf]

A

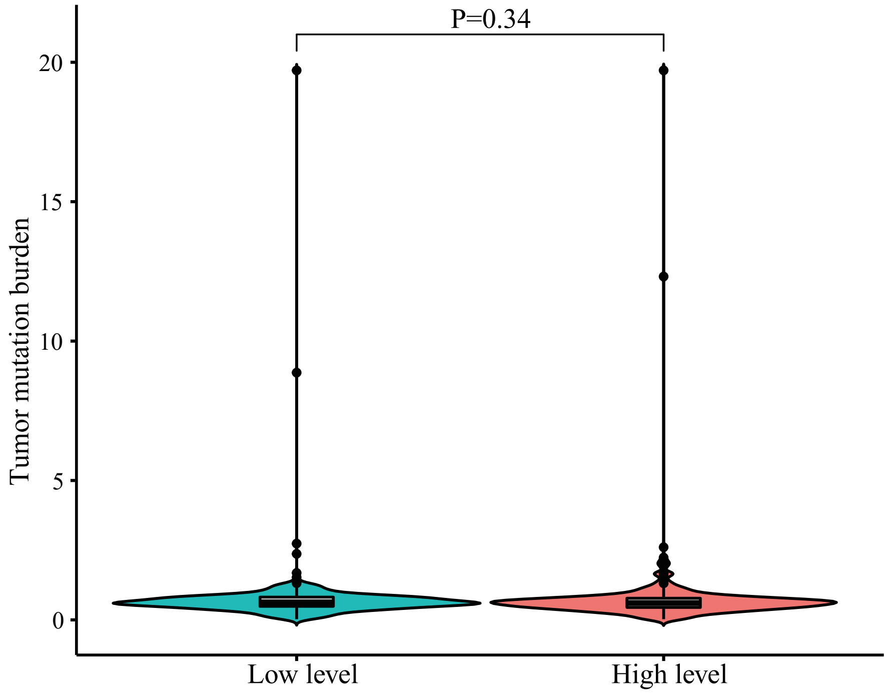

B

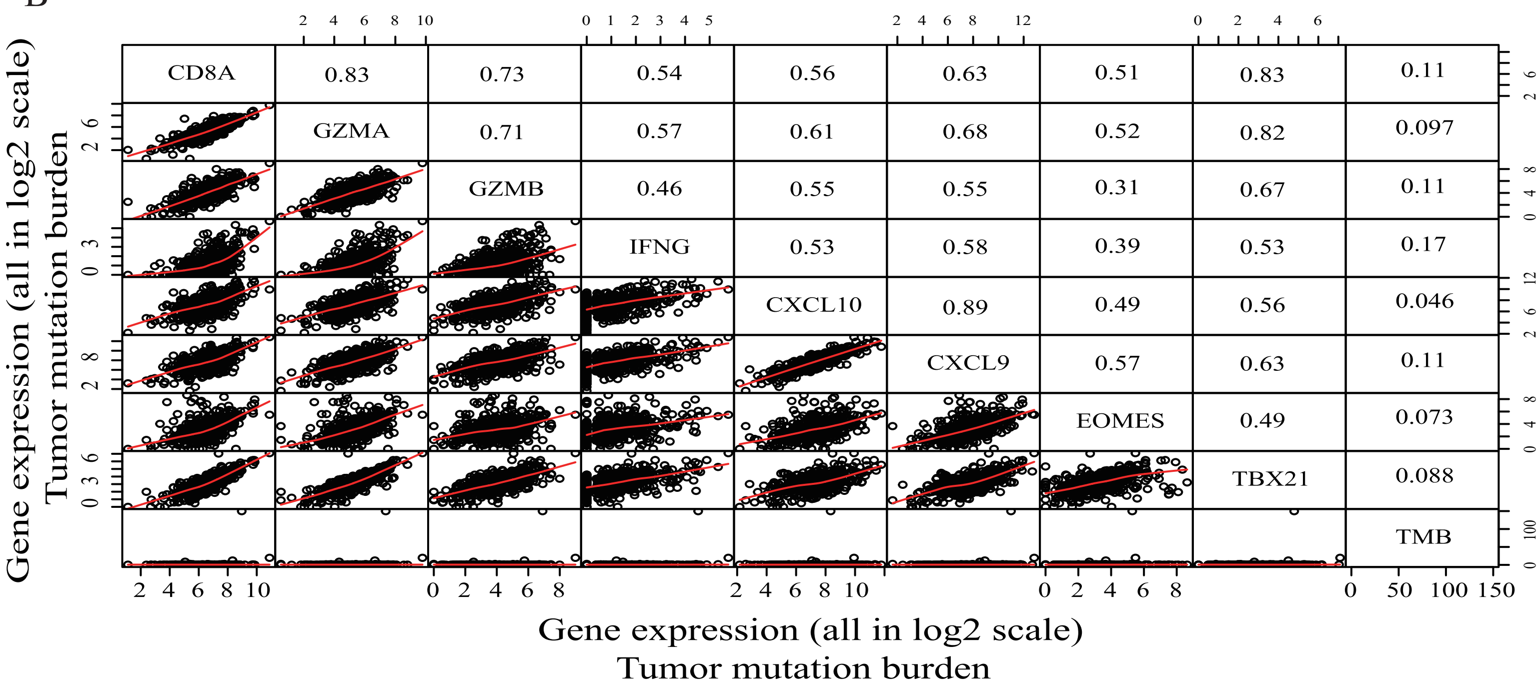

Supplementary Fig. 5 (A) The level of tumor mutation burden between the two clusters. Statistics were based on two-tailed Mann–Whitney U-test. **(B)** Correlation of expression level of anti-PD-1-responsive gene panels (CD8A, GZMA, GZMB, IFNG, CXCL10, CXCL9, EOMES and TBX21, defined by POPLAR trial previously) with tumor mutation burden.
