# Supplementary material for: Immune infiltration phenotypes of prostate adenocarcinoma and their clinical implications
Source: Cancer Med. 2021 Jun 15;10(15):5358–74. doi: 10.1002/cam4.4063 (PMC8335836; doi:10.1002/cam4.4063)
Supplement: Supplementary file 6 — Fig S6 [file CAM4-10-5358-s009.pdf]

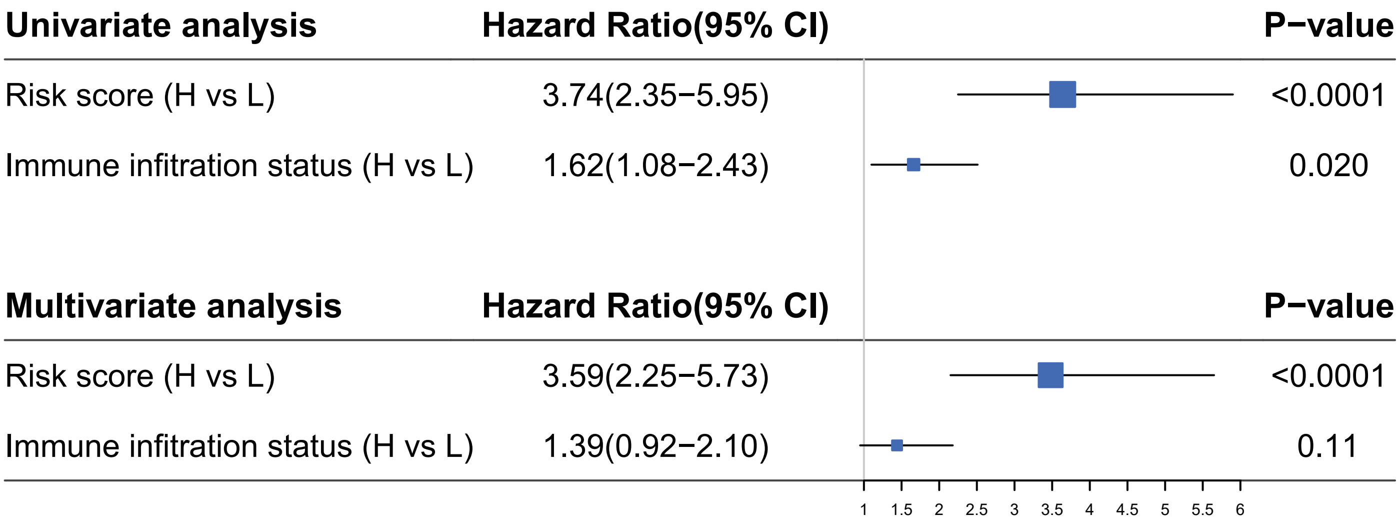

Supplementary Fig. 6 Univariate and multivariate regression analysis of the correlation between the IPM and the immune infiltration phenotypes associated with the DFS in the TCGA cohort.
